# Prevalence and Clustering of Lifestyle Risk Factors for Chronic Diseases Among Middle-Aged Migrants in Japan

**DOI:** 10.3390/healthcare13212781

**Published:** 2025-11-02

**Authors:** Hansani Madushika Abeywickrama, Yu Koyama, Mieko Uchiyama, Akiko Okuda

**Affiliations:** Graduate School of Health Sciences, Niigata University, 2-746, Asahimachi-dori, Chuo-ku, Niigata-shi, Niigata 951-8518, Japan; yukmy@clg.niigata-u.ac.jp (Y.K.); uchiyama@clg.niigata-u.ac.jp (M.U.); okudaa@clg.niigata-u.ac.jp (A.O.)

**Keywords:** non-communicable diseases, risk factors, clustering, migrants, Japan, latent class analysis

## Abstract

Background/Objectives: Migrants are recognized as a vulnerable population for non-communicable diseases (NCDs) due to unique socio-cultural and environmental challenges associated with acculturation. Despite a growing migrant population, evidence on NCD risk among migrants in Japan is scarce. This study examined the prevalence, co-occurrence, and clustering of modifiable NCD risk factors among middle-aged foreign residents in Japan. Methods: A cross-sectional web-based survey was conducted among foreign residents aged 30–60 years (*n* = 384). Eight risk factors were assessed: tobacco use (including cigarettes, e-cigarettes, and chewable tobacco), harmful alcohol consumption, inadequate fruit and vegetable intake, frequent consumption of ultra-processed foods, insufficient physical activity (PA), poor sleep, high stress levels, and high BMI. Latent class analysis (LCA) was used to identify risk clusters, and associations with demographic and socioeconomic characteristics were examined. Results: The prevalence of risk behaviours was high, with 96% reporting inadequate fruit and vegetable intake, 55% poor sleep, and 50% insufficient PA. Risk factor co-occurrence was common: 32% reported three concurrent risks, 27.1% reported four, and 17.2% reported five or more. LCA identified two clusters. Cluster 1 (46.9% of participants) was characterized by tobacco and alcohol use, with inadequate diet and poor sleep. Cluster 2 (53.1% of participants) was defined by insufficient PA, inadequate diet, and poor sleep, but low tobacco and alcohol use. Cluster membership varied significantly by sex and employment. Conclusions: This study provides preliminary evidence of the high prevalence and clustering of modifiable NCD risk factors among middle-aged migrants in Japan. Findings highlight the need for comprehensive, multi-behavioral interventions tailored to migrant populations, while considering gender roles and occupational contexts.

## 1. Introduction

Non-communicable diseases (NCDs), or chronic diseases, include cardiovascular diseases, diabetes, cancers, and chronic respiratory diseases, and accounted for over 40 million deaths and 1.73 billion disability-adjusted life years (DALYs) worldwide [1,2]. Primarily influenced by behavioral, physiological, and genetic factors, chronic diseases are generally incurable but can be managed through early detection, lifestyle changes, and medical interventions [1,2]. Four behavioral factors, including tobacco use, harmful alcohol consumption, unhealthy diet, and insufficient physical activity, are recognized as key modifiable risk factors for NCDs [1]. Additionally, psychological stress [3] and poor sleep habits [4] have been independently associated with the development and progression of chronic diseases. Risk factors for chronic diseases often co-occur and are interrelated, and assessing the prevalence of isolated risk factors alone does not capture their co-occurrence within individuals [5]. Identifying clusters of risk behaviours that commonly co-occur is crucial for designing effective lifestyle interventions [6].

Migrants may face a disproportionate risk for NCDs due to socio-cultural, economic, and environmental challenges after migration compared to native populations. These factors include limited access to healthcare services due to language or administrative barriers, challenges associated with dietary acculturation, lack of support systems, acculturation-related stress, and social isolation [7]. Consistent with this, high incidence and prevalence rates of chronic diseases among migrants from Asian and African countries to high-income regions, particularly Europe, North America, and Oceania, have been extensively reported [8,9,10,11,12,13]. With globalization and increasing migration worldwide, evidence on the NCD burden among migrant populations is also emerging from other regions such as the Middle East [14,15] and Asia [16,17,18].

Japan has become a popular destination for migrants in recent years, with the foreign resident population reaching over 3.7 million in 2024 [19]. The largest groups of foreign residents are from Asian countries, with the top five being China, Vietnam, South Korea, the Philippines, and Nepal, while the proportion from Western countries remains relatively low [19]. The proportion of the foreign population in Japan is relatively low (around 3%) compared with North America, Europe, and Oceania [20]. This low proportion may amplify health risks, as evidence suggests that migrants face greater challenges when their share in the host population is small [21,22]. In addition, Japan’s health system and administrative processes are conducted predominantly in Japanese, which can create barriers for migrants with limited proficiency. Furthermore, Japan’s unique dietary culture and lifestyle offer a distinct context compared to Western settings. These differences highlight the need for country-specific evidence on NCD risk factors among migrants in Japan. Despite this, evidence remains limited.

Some small-scale studies among migrant subgroups in Japan have reported elevated NCD risks. For example, among middle-aged female immigrants (*n* = 35), 29% had hypertension and 29% had obesity [23], while a free health check of 41 immigrant workers with unstable employment found many had clinical indicators requiring medical consultation [24]. Similarly, in a study of Thai migrants (*n* = 28), 41.8% reported chronic diseases [22]. Despite their small sample sizes, these findings highlight the need for larger, population-based studies to better understand NCD risk patterns among migrants in Japan.

Given the challenges associated with acculturation, clustering of NCD risk factors among migrant populations is likely to be common. Although several studies have examined the prevalence of isolated risk factors for chronic diseases [9,10,25,26,27,28], few have evaluated their coexistence within migrant populations [29]. To our knowledge, no studies have explored the prevalence or co-occurrence of NCD risk factors among migrants in Japan. Addressing this gap, the present study investigated the prevalence and co-occurrence of eight NCD risk factors, including tobacco use, harmful alcohol consumption, insufficient fruit and vegetable intake, frequent consumption of ultra-processed food (UPF), physical inactivity, poor sleep habits, high stress level, and high body mass index (BMI), among middle-aged (30–60 years) foreign residents in Japan. These factors were selected because they are among the most widely recognized modifiable risk factors contributing to the global burden of NCDs [1,2], and have also been used in previous studies examining the clustering of health behaviors and NCD risks [29,30]. We selected this age group due to several reasons, including (1) metabolic risk factors for NCDs such as hypertension, dyslipidaemia, and glucose intolerance, often begin to develop in the early 30 s and become clinically significant by 40 s [31,32,33]; (2) this life stage is marked by career development, family responsibilities, and increased psychosocial stress, which often trigger establishment of risk behaviours [34,35]; and (3) after the age of 60 many individuals are already diagnosed with or receiving treatment for chronic diseases, making the 30–60 age range suitable for early risk detection.

## 2. Materials and Methods

### 2.1. Study Design and Population

A web-based questionnaire survey was conducted in July 2025 using ‘Freeasy,’ a web research service provided by an internet survey company with approximately 13 million registered monitors in Japan (iBRIDGE Ltd., Tokyo, Japan). First, a screening survey was conducted among 50,000 participants aged 30–60 years, using the following four questions: (1) Were you born and raised in a foreign country? (2) Have you been living in Japan for more than three consecutive months? (3) Do you follow a prescribed therapeutic diet due to a medical condition? and (4) Are you pregnant (if applicable)? The screening survey was administered using quota settings to ensure balanced sampling.

The survey was distributed to 574 individuals who answered ‘yes,’ ‘yes,’ ‘no,’ and ‘no’ to the screening questions, respectively. The survey had a three-week response period, and a total of 400 responses were obtained. After excluding responses that were illogical, demonstrated non-differentiation (straightlining), or contained more than 20% missing data, a total of 384 valid responses were retained for analysis. A sample size of 384 was considered adequate, assuming an estimated prevalence of risk factors for NCDs at 50% due to the lack of prevalence data, a margin of error of 5%, and a confidence interval 95% [36]. However, as this was a convenience sample recruited through a web panel, certain migrant groups, such as those with limited internet access or lower literacy, may have been underrepresented.

### 2.2. Ethical Considerations

This study obtained the approval of the ethical committee of Niigata University, Japan (Approval No. 2025-0067). Informed consent was obtained through the web-based system before proceeding to the questionnaire. The study was conducted in accordance with the Declaration of Helsinki of the World Medical Association and reported according to the Strengthening the Reporting of Observational Studies in Epidemiology (STROBE) guidelines for cross-sectional studies (Table S1) [37].

No personally identifiable information was collected during the survey, which was designed to ensure complete respondent anonymity. The handling of personal information by iBRIDGE Ltd. is governed by its internal privacy policy, and the platform operates under robust data protection standards. The final dataset provided to the research team included only anonymized respondent IDs, which could not be used to identify individuals.

### 2.3. Data Collection

A questionnaire was developed based on the WHO STEPS survey instrument [38] and the FANTASTICO lifestyle questionnaire [39], which are widely used to assess NCD risk factors. Expert input from community medicine and clinical medicine was incorporated to improve the content validity of the questionnaire. The questionnaire, initially developed in English, was translated into Japanese using a forward-backward translation process by a bilingual expert familiar with public health terminology. English and Japanese versions were chosen as they are the most commonly used languages in academic, administrative, and healthcare settings in Japan. Both English and Japanese questionnaires were pilot tested with 10 foreign residents from diverse backgrounds, representing East Asia (*n* = 2), South Asia (*n* = 2), Africa (*n* = 2), the Americas (*n* = 2), and Europe (*n* = 2), to assess clarity and comprehension across different migrant groups. The pilot participants had varied educational levels, ranging from high school to graduate degrees, and included both students and full-time employees. Feedback from the pilot informed minor revisions in wording and structure to improve clarity.

Data on the characteristics of the respondents (age, gender, residential area, marital status, occupation, and annual income) were obtained from the registration information of the internet survey company. The questionnaire consisted of the following items.

- Socio-demographic data (education level, living conditions)
- Health-related data (having had a health screening, last time having a health screening, having been diagnosed with a chronic disease, on medication for any chronic disease, family history of chronic diseases)
- NCD risk factors
  - tobacco use (Cigarettes, e-cigarettes, chewable tobacco)
  - alcohol consumption
  - dietary habits:
    1. consumption and frequency of fruit and vegetables
    2. consumption of UPF
    3. consumption of excess salt, sugar, and fat
    4. type of oil used in cooking
  - engaging in PA
    1. moderate (activities that cause moderate increases in breathing or heart rate, such as brisk walking, lifting light loads, general cleaning, cycling, etc.) PA
    2. vigorous (activities that cause large increases in breathing or heart rate, such as sports, fitness, carrying or lifting heavy loads, climbing stairs, digging, construction work, etc.) PA
  - stress:
    1. frequency of stress
    2. sources of stress
    3. methods to relieve stress
    4. perceived stress level
  - sleep
    1. duration of sleep at night
    2. sleep disturbance
  - self-reported anthropometric data
    1. weight
    2. height
    3. waist circumference (WC)

The definitions of the eight ‘at-risk’ variables used in the cluster analysis are summarized in Table 1. While prevalence of high WC was also assessed, it was not included as a variable in the LCA due to two main reasons. First, WC is not typically treated as an independent risk factor when high BMI is already included, as both indicators capture overlapping aspects of adiposity and metabolic risk [40]. As the inclusion of both measures could have introduced redundancy into the LCA, BMI was selected as the primary anthropometric indicator to include in the LCA. Second, a considerable proportion of data on WC was missing, which could have reduced the overall sample size and compromised the robustness of the model. High WC was defined according to established cut-offs. Increased risk was defined as WC > 94 cm in men and >80 cm in women, while substantially increased risk refers to WC > 102 cm and >88 cm in women [40].

### 2.4. Data Analysis

Descriptive statistics were used to determine the prevalence and co-occurrence of NCD risk factors. Representative values are shown as numbers and percentages or mean and standard deviation. Missing values were observed only for WC, likely because many respondents did not know their measurements. Because imputing these values was considered unreliable, the actual number of responses was used to calculate percentages. The chi-square test and Mann–Whitney U test were performed to compare groups. Statistical significance was set at *p* < 0.05. Statistical analyses were performed using IBM SPSS Statistics version 25.0 (IBM Corporation, Armonk, NY, USA). Heatmaps were created using R Studio.

Latent class analysis (LCA) was conducted in R Studio to identify clusters of NCD risk factors (tobacco use, harmful alcohol consumption, low fruit and vegetable intake, UPF consumption, low PA, poor sleep, high stress, and high BMI). LCA models are parameterized by: (1) the assumed number of latent classes, (2) the proportion of participants in each class (class membership probabilities), and (3) the item-response probabilities for each risk factor within classes. Model parameters were estimated using the expectation–maximization algorithm. Models specifying two to ten classes were evaluated and compared using fit indices, including the Bayesian Information Criterion (BIC) and Akaike Information Criterion (AIC), as well as consideration of the interpretability of the resulting clusters. The optimal number of classes was selected according to the lowest BIC and interpretability of class profiles. Both AIC and BIC values were lowest for the two-class model (AIC = 3320.0; BIC = 3387.1), which was therefore selected as the optimal solution (Table S2). Item-response probabilities were examined to characterize each cluster, and participants were assigned to the class with the highest posterior probability of membership.

## 3. Results

### 3.1. Characteristics of the Survey Respondents

The mean age of the respondents was 44.72 (8.05), and 55.2% of them were male. Most of the participants were residents of the Kanto region, followed by the Kansai region and the Chubu region. Most of them were company employees with college or university education. Most participants reported an annual household income of 5,000,000–9,999,999 yen and were living with their families. Most participants had undergone health screening, with 61.2% screened within the past year. High blood pressure and high cholesterol were the commonly reported conditions, followed by diabetes and heart disease. Among those who were diagnosed with each condition, 56.5%, 38.8%, 59.3%, and 31.8% were currently on medication, respectively (Table 2).

### 3.2. Prevalence of NCD Risk Factors

#### 3.2.1. Tobacco and Alcohol Use

Current use was most common for alcohol, followed by cigarettes and e-cigarettes, while the majority had never used e-cigarettes or chewing tobacco (Table 3). Long-term use (>5 years) was most frequently reported among current users of alcohol, cigarettes, and e-cigarettes. Daily consumption patterns varied: most alcohol users consumed 1–2 drinks per day, whereas cigarette and e-cigarette users showed a relatively higher proportion of heavy use (Table S3). Among current users, heavy exposure was most frequent for current users of cigarettes and e-cigarettes, and among ex-smokers.

**Figure 1:**
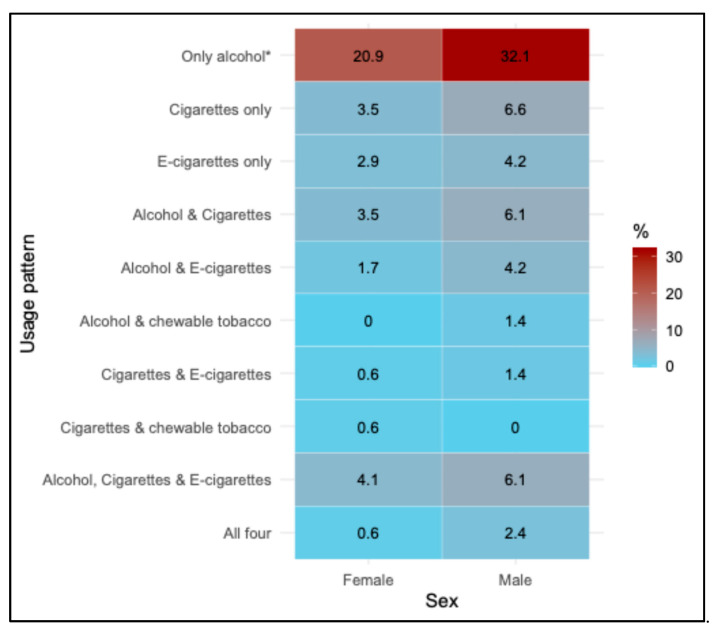
Heatmap of current use of tobacco and alcohol patterns by sex. Values represent percentages of females (*n* = 172) and males (*n* = 212) within each usage pattern category. A statistically significant difference between sexes was observed only for the ‘Only alcohol’ category based on the Chi-square test, as indicated by an asterisk (*p* < 0.05).

Sex-specific patterns of current use are presented in Figure 1. A significant sex difference was observed for the ‘only alcohol’ category, while men more frequently reported dual or multiple product use, such as alcohol with cigarettes or e-cigarettes, with no statistically significant difference (Table S4).

#### 3.2.2. Fruit and Vegetable Consumption

Daily consumption of fruits and vegetables was relatively low, with only 18.5% reporting eating fresh fruits and 25.0% consuming green vegetables every day. Legume and root/ tuber vegetable consumption was less frequent, with around 10–12% reporting daily intake. The majority reported moderate intake (3–4 days per week) across all categories. In terms of servings, nearly one-third of participants consumed less than one serving per day across all food groups (Table S5).

**Figure 2:**
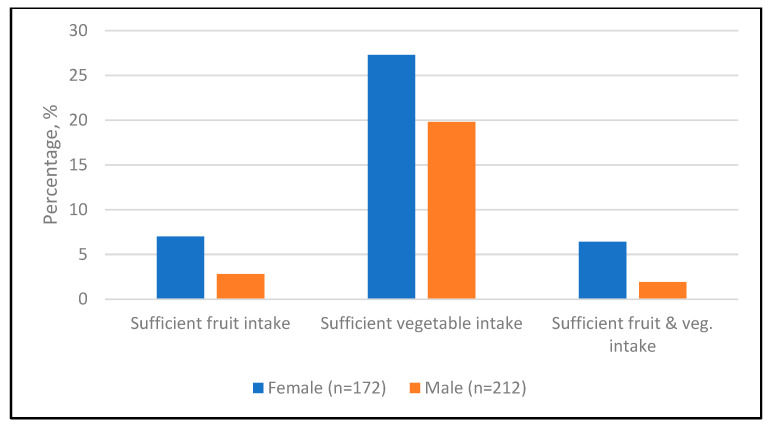
Adequacy of fruit and vegetable intake by sex. Values represent percentages of participants who met the recommended intake of fruits (≥2 servings) and vegetables (≥3 servings) within each sex.

Sufficient intake of fruit (4.7%) was lower compared with sufficient vegetable intake (23.2%), and adequate consumption of both fruits and vegetables (3.9%) was particularly low (Table S6). Across all categories, adequacy was consistently higher among females than among males, while a significant sex difference was observed only for combined fruit and vegetable intake (*p* = 0.045) (Figure 2).

#### 3.2.3. Consumption of Ultra-Processed Food

Processed salty foods (such as chips, salted nuts, canned foods such as stews or vegetables, pickles, etc.), sugar-sweetened beverages (soft drinks, energy drinks, etc.), sweet snacks (cakes, cookies, candy, desserts, etc.), deep-fried foods (fried potato, fried chicken, tempura, etc.), and processed high-fat foods (sausage, bacon, butter, etc.) were mainly consumed 1–2 times per week (Table S7).

Females were more likely than males to consume sweet snacks frequently (*p* = 0.0013). Similarly, females reported a higher frequency for the combined categories of sugar-sweetened beverages or sweet snacks (*p* = 0.014) and sugar-sweetened beverages and sweet snacks (*p* = 0.029). In contrast, no significant differences were observed for processed-salty foods, deep-fried foods, and processed high-fat foods. Co-consumption patterns showed that approximately one-fourth of participants reported frequent intake of at least three UPF categories (Table 4).

**Figure 3:**
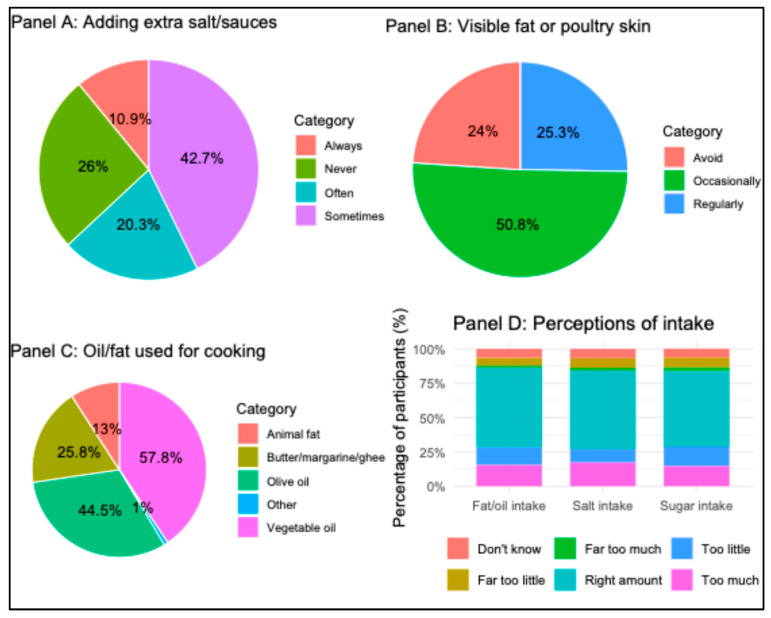
Perceptions and practices related to dietary salt, sugar, fat/oil intake among participants (*n* = 384). **Panel A:** Frequency of adding extra salt or salty sauces to food before or during eating. **Panel B:** Consumption of visible fat on meat or the skin on poultry. **Panel C:** Type of oil or fat commonly used during cooking. **Panel D:** Perceptions on the amount of consumption of fat/oil, salt, and sugar.

About 30% of the participants reported that they add extra salt or salty sauces before or during eating. Visible fat on meat or meat skin was consumed regularly by one fourth of the participants. Most participants perceived their intake of salt, sugar, and fat/oil as the ‘right amount,’ while smaller proportions reported excessive or insufficient intake. Vegetable oil and olive oil were the most commonly used for cooking (Figure 3).

#### 3.2.4. Physical Activity

Table 5 presents the proportion of participants engaged in vigorous PA, moderate PA, and sports or fitness, as well as the frequency and duration of these activities. Over half of the participants engaged in moderate activity, while about 40% reported doing sports, and about 30% reported doing vigorous activity. Most participants practiced physical activity 3–5 days per week, with moderate activity being the most frequent. Further, most of those who were doing sports or fitness were doing so once a week. In terms of duration, 30–59 min was the most common across all activity types.

Men were significantly more likely to engage in vigorous PA (41.0% vs. 25.6%, *p* = 0.001) and sports/fitness activities (46.7% vs. 34.3%, *p* = 0.014) compared with women (Table S8). The percentage of participants who achieved adequate PA (>150 min/week moderate PA, >75 min per week vigorous PA, or an equivalent combination) was 195 (50.8%). Among them, 120 (61.5%) were male, a significantly higher proportion compared with female (*p* = 0.011).

#### 3.2.5. Sleep and Stress

The mean sleep duration among participants was 6.31 (1.46) hours. More than half of the participants reported shorter sleep times (53.1%), while about 3% reported longer sleep times (>9 h). Sleep disturbance, defined as self-reported difficulty falling asleep or maintaining sleep, was relatively uncommon, with 24.2% reporting it ‘sometimes’ and 17.2% reporting it ‘often’ (Table 6).

The mean self-rated stress level was 4.06 (SD-3.37), with similar levels observed in men (4.12, SD-3.39) and women (3.98, SD-3.35). About 18.2% of participants reported feeling stressed ‘often’, and 48.7% reported feeling stressed ‘sometimes’. Among those who reported stress often or sometimes, 43.6% rated their stress intensity as moderate, while 42.7% rated it as high. Work and finance were the most common sources of stress, while women additionally cited family and health-related stress. The most common coping strategies included sleep, talking to someone, and exercise, with notable sex differences. For example, females were more likely to speak to someone, while males were more likely to use exercise. Among those who reported feeling stressed often, 74.3% had high self-reported stress levels (Table 6).

#### 3.2.6. High BMI and Abdominal Obesity

The median BMI and WC were significantly higher in males compared to females. Based on BMI classification, the majority of participants were within the normal weight range; however, underweight was more common in females, while overweight and obesity were more prevalent in males. In our sample, the combined prevalence of overweight (13.4%) and obesity (30.7%) was lower than national GBD 2021 estimates for Japanese adults (26.4% in females and 34.4% in males), although the sex-specific pattern of higher rates among men remained consistent [50]. A higher proportion of females were classified as having increased or substantially increased risk by WC (Table 7).

### 3.3. Co-Occurrence of NCD Risk Factors

Table 8 illustrates the accumulation of multiple risk factors for chronic diseases among study participants by sex and age group (Table 8). Among females, the majority reported 2–4 concurrent risk factors, particularly in the 50–60 age group. Few women reported 5 or more concurrent factors. Among males, clustering of three to four risk factors was most common. In younger males, 44.3% had three risk factors, while the burden shifted towards 4 or more risk factors with increasing age. Notably, 27.1% of older men (50–60 years) exhibited clustering of 5 or more risk factors. Clustering of NCD risk factors appeared to be patterned by employment, education, and income, with individuals from lower education levels and low-to-middle income households disproportionately represented in higher co-occurrence groups, although these associations were not statistically significant. Higher co-clustering was observed among migrants diagnosed with chronic conditions such as hypertension, diabetes, and high cholesterol (Table S9).

Clustering of multiple risk factors was common in the total population. Approximately one-third of participants (32%) reported 3 concurrent risk factors, while 27.1% reported 4 risk factors, and 17.2% exhibited 5 or more co-occurring risks. The presence of only a single risk factor was relatively rare, observed in only 18 participants (4.7%).

### 3.4. Clustering of NCD Risk Factors

**Figure 4:**
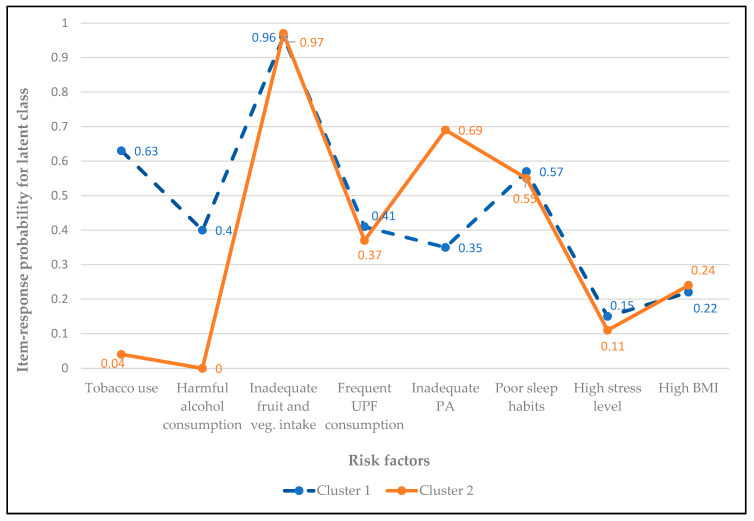
Item-response probabilities for NCD risk factors by latent class. Cluster 1 (46.9% of participants) represents the ‘tobacco/alcohol-diet cluster,’ characterized by higher probabilities of inadequate fruit and vegetable intake, tobacco use, poor sleep, and harmful alcohol consumption. Cluster 2 (53.1% of participants) represents the ‘sedentary life-diet cluster,’ defined by low probabilities of tobacco and alcohol use but high likelihood of physical inactivity, inadequate fruit and vegetable intake, and poor sleep. Abbreviations: BMI—Body mass index, PA—Physical activity, UPF—Ultra-processed foods.

Two clusters were selected as optimal based on the interpretability of the identified clusters in the LCA. Cluster 1 (46.9% of participants) was characterized by higher probabilities of inadequate fruit and vegetable intake (0.96), tobacco use (0.63), poor sleep (0.57), and harmful alcohol consumption (0.40). In contrast, cluster 2 (53.1% participants) was marked by low tobacco and alcohol use, but higher probabilities of inadequate fruit and vegetable intake (0.97), inadequate PA (0.69), and poor sleep (0.55). These findings suggest the presence of two distinct NCD risk factor clusters: ‘tobacco/alcohol-diet cluster,’ and ‘sedentary life–diet cluster’(Figure 4).

The cluster membership varied significantly by sex and employment status. Men and full-time employees were more likely to belong to the tobacco/alcohol-diet cluster, whereas women and housewives were more likely to belong to the sedentary life-diet cluster. Other socio-economic parameters, including age, education, income, and living conditions, were not significantly associated with latent class membership. The majority of the participants diagnosed with chronic diseases, such as hypertension, diabetes, high cholesterol, and heart disease, were concentrated in the tobacco/alcohol-diet cluster (Table 9).

## 4. Discussion

This study provides an overview of the prevalence, co-occurrence, and clustering of risk factors for chronic diseases among middle-aged foreign residents in Japan. Among the risk factors observed, the most prevalent one was inadequate fruit and vegetable consumption (96%), followed by shorter or longer sleep duration (56.3%), and physical inactivity (49.2%). The findings reveal that a substantial proportion of survey respondents had multiple risk factors (95.3%), rather than a single risk factor. Two risk clusters were identified: cluster 1, tobacco/alcohol-diet cluster, and cluster 2, sedentary life–diet cluster. Sex and employment status were associated with the cluster membership.

### 4.1. Prevalence of Risk Factors

The current findings showed that alcohol consumption was a common habit among survey participants, with most of them reporting consuming 1–2 drinks per day for more than 5 years. While earlier studies suggested an association between low or moderate alcohol intake and a protective effect against specific chronic conditions such as IHDs and type 2 diabetes [51,52], recent and comprehensive evidence emphasizes that any level of alcohol consumption contributes to health loss, including increased risk of cancers and all-cause mortality [53]. In the present study, harmful drinking was defined as a daily intake of ≥3 alcoholic drinks, a threshold commonly used in assessing chronic disease risk. However, if any amount of alcohol were to be considered harmful, as suggested by the GBD study [53], the proportion of participants at chronic disease risk would be substantially higher.

On the other hand, although most of the participants have never used cigarettes, e-cigarettes, or chewable tobacco, both current and former users demonstrated long-term and heavy daily use, suggesting that these behaviors are not merely transient but may accumulate into substantial long-term health risks. Tobacco use is a well-established determinant of NCDs, particularly cardiovascular and respiratory diseases, and tobacco-related deaths are projected to continue rising globally despite ongoing control efforts [54]. In the current study, simultaneous use of alcohol and tobacco was also present, more commonly among men. Previous studies also suggest that tobacco and alcohol consumption frequently coexist [55] and synergistically amplify health risks [56].

Dietary risk factors were highly prevalent among foreign residents in this study, as reflected in the inadequate consumption of fruits and vegetables, alongside a frequent intake of UPF, added salt, and visible fat. These behaviors reflect the cumulative effects of the nutrition transition and dietary acculturation frameworks. The nutrition transition is characterized by the global shift toward increased consumption of ultra-processed foods that are typically high in sodium, sugar, saturated fats, and refined carbohydrates, which are strongly associated with obesity, hypertension, and type 2 diabetes [57]. In parallel, dietary acculturation, the process by which migrants change or adapt their diet to the host country’s food environment and cultural norms, has been identified as a critical contributor to chronic disease risk among migrant populations in developed countries [58]. For middle-aged foreign residents in Japan, these dietary habits may be associated with increased reliance on convenience foods, fast food chains, and packaged meals. Moreover, barriers such as limited access to culturally familiar foods, cost of fresh fruits and vegetables, and time constraints due to work schedules may further reinforce these unhealthy dietary patterns [59].

In the current study, over half of the participants engaged in moderate PA, more than 40% participated in sports, and approximately half met the WHO-recommended thresholds for weekly PA. In our previous study, over 40% of respondents reported an increase in PA levels after migrating to Japan [59], suggesting that migration-related lifestyle changes may have contributed to the relatively high participation in PA observed in the present study [59]. Significantly lower PA levels have been reported among migrants, attributable to barriers such as changes in the physical and cultural environment, lack of culturally sensitive services, and limited familiarity or comfort in accessing facilities in the host country [60,61].

In the current study, shorter sleep duration was reported by over half of the respondents, which is a concerning finding given the strong association between insufficient sleep and metabolic risk. Beyond sleep duration, other dimensions such as sleep stages and regularity have also been linked to the development of chronic diseases [4]; however, these aspects were not assessed in the present study. Although high stress was not widely prevalent, work-related stress was the most commonly cited source among both sexes, while female participants additionally reported family- and health-related stress. The absence of extended family networks and limited access to culturally appropriate support systems for childcare and household responsibilities may have contributed to both insufficient sleep and higher stress levels among women. Migrants often experience acculturation stress, exacerbated by factors such as challenging living conditions, cultural and language barriers, social isolation, and a feeling of disconnection from home. These factors may lead to maladaptive coping behaviors, such as smoking and harmful alcohol use, reliance on convenient dietary patterns and sedentary lifestyles [35]. Additionally, structural barriers such as legal status, unfamiliarity with the healthcare and welfare systems, and limited language proficiency may restrict access to preventive services. These constraints can amplify the risk of NCDs among migrants, underscoring the importance of addressing systemic as well as individual-level factors in migrant health strategies.

Anthropometric analysis revealed clear sex differences. While more than 60% of the participants were within the normal BMI range, men were more likely to be overweight or obese. A recent meta-analysis has estimated the global prevalence of overweight and obesity at 37% and 24% among immigrant populations worldwide. When stratified by sex, prevalence rates of overweight and obesity were 20% and 44% among men, and 34% and 27% among women, respectively. In Asia, pooled estimates indicated a prevalence of 33% for overweight and 23% for obesity [62]. Compared to these global and regional statistics, the prevalence rates in the current study are substantially lower.

Various factors may explain these differences. First, Japan has one of the lowest national obesity rates among high-income countries, attributed to dietary patterns, active lifestyle, etc. Migrants residing in Japan may adopt or be influenced by these healthier practices, thereby lowering their risk compared to migrant populations in other high-income countries. Second, underreporting and misclassification cannot be excluded, as BMI alone does not fully capture body composition. Notably, abdominal obesity among migrant women was higher despite lower overall BMI in this study, consistent with prior evidence suggesting that reliance on BMI alone may underestimate cardiometabolic risk. Additionally, psychosocial and structural factors are likely to influence these anthropometric outcomes. For instance, migrants in Japan often face barriers to maintaining a healthy diet, including affordability, limited access to culturally familiar foods, and a reliance on convenience or processed meals due to time constraints [59,63]. Furthermore, factors such as restricted calorie intake due to body image concerns, irregular portion sizes, or eating schedules resulting from employment conditions like physically demanding jobs and shift-based work, also affect these observations.

### 4.2. Co-Occurrence and Clustering of Risk Factors

Most participants in our study had multiple concurrent risk factors, which substantially magnified the risk of chronic disease comorbidity and premature mortality [64,65]. The high rates of concurrent risk factors observed in our study appear greater than the reported prevalence rates among general adult populations worldwide [66,67,68]. These variations in prevalence may be partly explained by differences in the number and type of risk factors considered, as well as the sociodemographic and occupational characteristics of the populations studied. Importantly, unlike these studies that primarily examined relatively homogeneous national populations, our sample consisted of foreign residents of different nationalities. This heterogeneity, combined with migration-related factors mentioned previously, may also have contributed to the higher prevalence of co-occurring risk factors observed.

This study identified two distinct clusters of NCD risk factors among middle-aged foreign residents in Japan. Cluster 1 (tobacco/alcohol-diet cluster) was tobacco use, harmful alcohol consumption, inadequate fruit and vegetable intake, and poor sleep, while cluster 2 (sedentary life-diet cluster) was defined by high physical inactivity alongside a similarly poor diet and sleep quality. These findings further emphasize that risk behaviours do not occur in isolation but co-occur in identifiable patterns that warrant targeted public health interventions.

Clusters defined by tobacco and alcohol use have been consistently identified in several previous studies [6,64,69,70]. Notably, however, the probability of tobacco use in Cluster 1 in our study (0.63) was higher than that reported previously [6,65,66]. This difference may be partly explained by our broader definition of tobacco use, which included e-cigarette use and tobacco chewing, as well as our consideration of heavy exposure among former users, factors often overlooked in earlier studies. Cluster 2 exhibited probabilities greater than 0.5 for inadequate fruit and vegetable intake, insufficient physical activity, and poor sleep quality. The co-occurrence of dietary risk and physical inactivity has also been reported in prior research, emphasizing the tendency of lifestyle-related risks to cluster [6,68,71,72]. However, direct comparisons across studies remain difficult due to differences in study populations, tools used to measure risk factors, and the specific risk factor combinations analysed.

Cluster membership was not evenly distributed across the population but varied significantly by sex and employment. Men were more likely to belong to the tobacco/alcohol-diet cluster (68.3% of men), consistent with global evidence of higher tobacco and alcohol use among males [73,74]. In contrast, women and housewives were more likely to belong to the sedentary life-diet cluster (66.9% of women), reflecting broader trends of sedentary behavior described in previous studies, where women typically engage in less leisure-time activity and lower intensity activity compared with men [75]. These patterns are consistent with our findings from Table S8, which showed that men were significantly more likely to participate in vigorous PA, sports/ fitness, and to meet adequate PA guidelines.

Employment status also differentiated the clusters: full-time company and government employees were overrepresented in the tobacco/alcohol-diet cluster, suggesting occupational and social environments that may facilitate tobacco and alcohol use [73,74]. Workplace culture and full-time work have been consistently linked with higher rates of smoking and alcohol consumption, particularly in Japan, where ‘*nomikai*’ (after-work drinking parties) are deeply integrated into workplace culture and often reinforce these behaviors [76,77,78]. Importantly, the burden of chronic medical conditions was disproportionately higher in the tobacco/alcohol-diet cluster, with significantly greater prevalence of hypertension, diabetes, high cholesterol, and heart disease. This pattern suggests that tobacco- and alcohol-related risks may exert more immediate and severe metabolic consequences than sedentary lifestyle risks, although both clusters contribute substantially to long-term health implications.

According to GBD 2021, smoking and high alcohol use rank among the top five behavioural risk factors for death and disability worldwide. Dietary risk factors, such as low intake of fruits/vegetables and high consumption of processed foods, are consistently among the leading risk contributors to global disease burden [74]. Available evidence reinforces our observation that substance use and poor diet, when clustered, represent harmful profiles that demand urgent intervention. Therefore, identifying migrants who belong to cluster 1 should be performed through screening as a high-risk group, warranting priority in targeted prevention and management interventions.

### 4.3. Implications for Practice and Research

Our findings provide preliminary evidence of the clustering of modifiable behaviors in the migrant population in Japan, underscoring the need for integrated health promotion strategies that address multiple risk factors simultaneously. The two clusters identified suggest distinct targets for intervention and policy. Cluster 1 (tobacco/alcohol-diet cluster) highlights the need for culturally tailored health promotion programmes that integrate smoking cessation, alcohol reduction, and nutritional counselling, particularly in workplace settings with a high proportion of male migrant full-time employees. Cluster 2 (sedentary lifestyle–diet cluster) requires strategies to promote PA, support sleep hygiene, and improve dietary habits, delivered through community- and family-based approaches tailored to women and those engaged primarily in caregiving or domestic roles. Together, these recommendations support integrated, gender- and context-specific approaches that address both behavioral and structural determinants of NCD risk among migrants.

Future research should build on these findings by incorporating biometric validation, such as biomarkers and clinical examinations, to complement self-reported behaviors and reduce reporting bias. In addition, qualitative or mixed-method research is needed to capture migrants’ cultural perceptions and lived experiences related to health behaviors, which may influence both the adoption of and barriers to lifestyle change. Comparative studies between migrant and native Japanese populations would further help to clarify the unique contributions of migration status to risk factor clustering. Studies employing a longitudinal design are necessary to better capture the dynamics of risk factor accumulation and their long-term health consequences. Moreover, interventional studies should be planned to evaluate the impact of tailored and culturally sensitive prevention and management strategies in real-world settings. Finally, future studies should include larger and more diverse migrant groups in Japan to improve generalizability. Together, these approaches would provide a more comprehensive understanding of behavioral risk patterns and inform the design of culturally sensitive and evidence-based interventions.

### 4.4. Strengths and Limitations

This study provides preliminary evidence to better understand NCD risk among migrant populations in Japan, for which empirical data remain scarce. By focusing on middle-aged adults, we targeted an at-risk population to support the development of preventive interventions. Furthermore, by examining multiple risk factors simultaneously, this study offers a more comprehensive assessment of risk patterns in this population.

However, several limitations should be noted. First, participants were recruited through a web-based survey panel, and this convenient sampling approach may have led to underrepresentation of migrants with limited internet access or lower literacy. Additionally, the questionnaire was available only in English and Japanese, which may have excluded migrants with limited proficiency in either language. Together, these factors may limit the generalizability of the findings to the broader migrant population in Japan. Second, all data were self-reported, which could have led to both over- and underestimation of actual risk behaviors. Third, the cross-sectional study design prevents causal inference, and the sample may not fully capture the diversity of all foreign resident groups in Japan.

## 5. Conclusions

The prevalence of NCD risk factors was relatively high among the middle-aged migrants in Japan, with substantial co-occurrence of multiple risk factors. Two distinct clusters were identified: one characterized by tobacco and alcohol use, and another by inadequate physical activity. Both clusters shared common features of inadequate fruit and vegetable intake and poor sleep habits.

These findings highlight the high prevalence of NCD risk factors among foreign residents in Japan, a population facing unique challenges such as acculturation stress, language barriers, and limited social support. To address these issues, interventions should adopt a comprehensive, multi-behavioral approach that is both culturally tailored and accessible, simultaneously targeting tobacco and alcohol use, physical inactivity, poor diet, and inadequate sleep. At the policy level, migrant-specific health promotion programmes and workplace- or community-based initiatives are needed to reduce the disproportionate burden of risk behaviors in this vulnerable population. Finally, future research should employ longitudinal designs and incorporate biomarker-based assessments to capture the dynamics of risk accumulation and validate self-reported data. Qualitative and comparative studies are also needed to understand cultural perceptions and to contrast migrants’ risk profiles with those of native Japanese populations.

## Figures and Tables

**Table 1:** Measures of NCD risk and definition of ‘risk’.

Risk Factor	Related Measures (Response Options Used in the Survey)	Definition of ‘Risk’
1. Tobacco use (Cigarettes, e-cigarettes, chewable tobacco)	Practice: Current, past, or never Among current and past users: Duration of use (<6 months, 6 months–<1 year, 1–5 years, >5 years) Units per day ^a^ (1–2, 3–5, 6–10, >10)	Current users: Any type and level of tobacco use [41]. Former risk exposure: ≥10 unit-years ^b^ [42]
2. Harmful alcohol consumption	Practice: Current, past, or never Among current and past users: Duration of use (<6 months, 6 months–<1 year, 1–5 years, >5 years) Units per day ^a^ (1–2, 3–5, 6–10, >10)	Current users: For women—2 or more drinks in a day For men—3 or more drinks in a day [43]. Former risk exposure: consumption of three or more drinks for more than 1 year ^c^.
3. Insufficient fruit and vegetable consumption (green vegetables, legumes, roots and tubers, and other vegetables) ^d^	Consumption frequency: Everyday, 5–6 days, 3–4 days, 1–2 days, once/week, rarely/never Number of servings per day ^e^: <1, 1, 2, 3, >4	Fruit intake: no daily consumption or consumption of <2 servings/day Vegetable intake ^f^: <3 servings/day across all vegetable categories and consuming on <5 days/week [44,45].
4. Frequent consumption of UPF ^g^	Consumption frequency: Everyday, 5–6 days, 3–4 days, 1–2 days, 2–3 times/ month, once/ month, rarely/never	Consumption of any type of UPF almost every day or 5–6 days/week [46].
5. Inadequate PA	Number of days per week engaged in moderate PA and/or vigorous PA for at least ten minutes at a time ^h^ [frequency]; Minutes spent engaged in vigorous and/or moderate PA ^h^ [duration]	<150 min weekly moderate activity ^i^ or, <75 min weekly vigorous activity ^i^ or, less than an equivalent combination of both ^j^ [6].
6. Poor sleep habits	Number of hours sleep at night	Individuals with shorter (≤6 h) or longer (>9 h) daily sleep duration [47]
7. High stress level	Frequency of stress (often, sometimes, rarely, never) Perceived stress level, ranging from 1 (lowest) to 10 (highest).	Feeling stressed ‘often’ and self-reported stress level ≥ 7 [48]
8. High BMI	Current weight (kg) Current height (cm)	BMI ≥ 25 kg/m^2^ (Overweight or obese) ^k^ [49]

^a^ Units per day were defined as follows: number of cigarettes smoked per day, number of sessions or puffs of e-cigarettes taken per day, number of times chewable tobacco is used per day, or number of alcoholic drinks or shots taken per day. ^b^ Former risk exposure was calculated based on units per day and duration (For example, 10 unit-years equals 10 units per day for one year, or 5 units per day for 2 years). As our questionnaire included categorical inputs, we used bin midpoints to approximate units (<5 = 2.5, 6–10 = 7.5, and >10 = 12.5), and duration (<1 year = 0.5 year, 1–5 years = 2.5 years, >5 years = 5 years). ^c^ One standard drink was assumed to contain approximately 10 g of pure alcohol. Although heavy drinking is defined as 4 or more drinks per day for women and 5 or more drinks per day for men [38], the categorical input of our questionnaire was 3–5 drinks. Therefore, for both men and women, daily consumption of 3 or more drinks for more than one year was considered heavy drinking for ex-drinkers. ^d^ ‘Roots and tubers’ refer to edible non-starchy roots (carrots, beets, radishes, turnips) and exclude starchy root crops such as potatoes, cassava, and yams. ‘Other vegetables’ include onions, eggplant, pumpkin, okra, and bell peppers, among others, which do not fall under the categories of ‘green vegetables’ (such as spinach, lettuce, kale, cabbage, and broccoli), ‘fresh legumes’ (like green beans, edamame, and snow peas), or ‘roots and tubers.’ ^e^ 1 count of serving refers to ‘1 cup of raw vegetables,’ ‘½ cup of cooked vegetables,’ ‘1 medium-sized fruit,’ and ‘1/2 cup of cut fruit.’ ^f^ Adequate vegetable consumption was defined as the intake of ≥5 days per week, totaling ≥ 3 servings/day across all vegetable categories, reflecting both quantity and consistency of dietary habits in line with nutritional recommendations [39,40]. ^g^ UPF refers to ultra processed foods and include processed salty foods (such as chips, salted nuts, canned foods such as stews or vegetables, pickles, etc.), sugary drinks (soft drinks, energy drinks, etc.), sweet snacks (cakes, cookies, candy, etc.), deep-fried foods (fried potato, fried chicken, tempura, etc.), high-fat processed foods (sausage, bacon, butter, etc.), and refined carbohydrates (white flour, white bread, sugary cereals, etc.) almost every day. ^h^ Moderate PA refers to activities that cause moderate increases in breathing or heart rate, such as brisk walking, lifting light loads, general cleaning, cycling, etc, and vigorous PA includes activities that cause large increases in breathing or heart rate, such as sports, fitness, carrying or lifting heavy loads, climbing stairs, digging, construction work, etc. ^i^ Weekly activity minutes were calculated by multiplying the reported number of days per week by the midpoint of the reported duration category. The following mid-points were used: <30 min = 15 min; 30–59 min = 45 min; 1–2 h = 90 min; >2 h = 150 min. ^j^ An equivalent combination of moderate nd vigorous PA was derived by dividing the total weekly minutes of moderate PA by two and adding that to the total weekly minutes of vigorous PA. A combined total of <75 min per week was classified as inadequate. ^k^ The BMI was calculated as weight (kg)/height (m)^2^.

**Table 2:** Demographic and health-related characteristics of the survey respondents (*n* = 384).

		*n*	%
Age	30–39 years	116	30.2
	40–49 years	149	38.7
	50–60 years	119	31.0
Sex	Male	212	55.2
	Female	172	44.8
Marital status	Married	234	60.9
	Unmarried	150	39.1
Residential region	Hokkaido	15	3.9
	Tohoku	20	5.2
	Kanto	165	43.0
	Chubu	62	16.1
	Kansai	84	21.9
	Chugoku	21	5.5
	Shikoku	9	2.3
	Kyushu and Okinawa	29	7.6
Employment status	Company employee (Full-time)	193	50.3
	Company employee (Contract)	27	7.0
	Part-time work	53	13.8
	Government employee	19	4.9
	Self-employee	13	3.4
	Housewife	31	8.1
	Business owner/ executive	10	2.6
	Doctor/ medical personnel	4	1.0
	Freelancer	9	2.3
	Unemployed	14	3.6
	Student	2	0.5
	Other	9	2.3
Annual household income	<1,000,000¥	19	4.9
	1,000,000–4,999,999¥	133	34.7
	5,000,000–9,999,999¥	158	41.2
	10,000,000–14,999,999¥	44	11.5
	15,000,000–19,999,999¥	18	4.7
	>20,000,000¥	12	3.1
Degree of Education	No formal schooling	6	1.6
	Primary/elementary school	6	1.6
	Secondary/junior high school	14	3.6
	High school	101	26.3
	College/university	194	50.5
	Postgraduate	60	15.6
	Other	3	0.8
Living condition	Living alone	100	26.0
	Living with spouse/partner	84	21.9
	Living with family	198	51.6
	Other	2	0.5
Health screening history	Yes	293	76.3
Time since last screening	A year ago	235	61.2
	2 years ago	21	5.5
	3 years ago	19	4.9
	4 or more years ago	18	4.7
Presence of medical conditions	High blood pressure	69	18.0
	Diabetes	27	7.0
	High cholesterol	67	17.4
	Heart diseases	22	5.7
Currently on medication	High blood pressure	39	10.2
	Diabetes	16	4.2
	High cholesterol level	26	6.8
	Heart disease	7	1.8
Family history	High blood pressure	81	21.0
	Diabetes	52	13.5
	High cholesterol level	51	13.2
	Heart disease	23	6.0

**Table 3:** Tobacco and alcohol use among participants.

		Cigarettes	E-Cigarettes	Chewable Tobacco	Alcohol
Practice (*n* = 384)	Current users	70 (18.2)	55 (14.3)	11 (2.9)	164 (42.7)
	Former users	109 (28.4)	77 (20.1)	44 (11.5)	90 (23.4)
	Never used	205 (53.4)	252 (65.6)	329 (85.7)	130 (33.9)
Heavy exposure *	Current users	52 (74.3)	42 (76.4)	1 (9.1)	59 (36.0)
	Former users	60 (55.0)	16 (20.8)	11 (25.0)	29 (32.2)

* Percentages were calculated based on the respective number of current or former users for each product.

**Table 4:** Prevalence of frequent UPF consumption by sex.

	Female (*n* = 172)	Male (*n* = 212)
Processed salty food	26 (15.1)	30 (14.2)
Sugar-sweetened beverages	44 (25.6)	41 (19.3)
Sweet snacks *	46 (26.7)	28 (13.2)
Sugar-sweetened beverages or sweet snacks *	62 (36.0)	51 (24.1)
Sugar-sweetened beverages and sweet snacks *	28 (16.3)	18 (8.5)
Deep-fried foods	20 (11.6)	23 (10.8)
Processed high-fat foods	27 (15.7)	27 (12.7)
Deep-fried or high-fat processed foods	32 (18.6)	38 (17.9)
Deep-fried and high-fat processed foods	15 (8.7)	12 (5.7)
Number of UPF categories consumed		
5	10 (5.8)	8 (3.8)
4	5 (2.9)	2 (0.9)
3	9 (5.2)	8 (3.8)
2	18 (10.5)	16 (7.5)
1	30 (17.4)	45 (21.2)
0	100 (58.1)	133 (62.7)

Data represent the numbers and percentages of participants reporting frequent consumption (defined as intake almost every day or 5–6 days per week) of each UPF category. Percentages were calculated within each sex category. Differences between sexes were calculated using the Chi-square test, with statistical significance set at *p* < 0.05 (marked by *).

**Table 5:** Physical activity habits among participants.

		Vigorous PA	Moderate PA	Sports/Fitness
Practice (*n* = 384)		131 (34.1)	199 (51.8)	158 (41.1)
Frequency *	Everyday	22 (5.7)	52 (13.5)	23 (6.0)
	6 days/week	14 (3.6)	13 (3.4)	10 (2.6)
	5 days/week	27 (7.0)	47 (12.2)	22 (5.7)
	4 days/week	16 (4.2)	19 (4.9)	26 (6.8)
	3 days/week	13 (3.4)	17 (4.4)	16 (4.2)
	2 days/week	16 (4.2)	25 (6.5)	20 (5.2)
	Once/week	23 (6.0)	26 (6.8)	41 (10.7)
Duration of PA *	<30 min	38 (9.9)	66 (17.2)	40 (10.4)
	30–59 min	46 (12.0)	74 (19.3)	60 (15.6)
	1–2 h	27 (7.0)	35 (9.1)	47 (12.2)
	>2 h	20 (5.2)	24 (6.3)	11 (2.9)

Data are presented as numbers and percentages. * Percentages were calculated based on participants who reported engaging in the respective type of physical activity.

**Table 6:** Sleep patterns and stress levels among participants.

	Total (*n* = 384)	Male (*n* = 212)	Female (*n* = 172)
Shorter sleep time (≤6 h)	204 (53.1)	112 (52.8)	92 (53.5)
Longer sleep time (>9 h)	12 (3.1)	3 (1.4)	9 (5.2)
Difficulty falling or staying asleep			
Often	66 (17.2)	37 (17.5)	29 (16.9)
Sometimes	93 (24.2)	45 (21.2)	48 (27.9)
Rarely	166 (43.2)	95 (44.8)	71 (41.3)
Never	59 (15.4)	35 (16.5)	24 (14.0)
Frequency of stress			
Often	70 (18.2)	40 (18.9)	30 (17.4)
Sometimes	187 (48.7)	101 (47.6)	86 (50.0)
Rarely	100 (26.0)	57 (26.9)	43 (25.0)
Never	27 (7.0)	14 (6.6)	13 (7.6)
Intensity of stress			
Low (0–3)	36 (9.4)	16 (7.5)	20 (11.6)
Moderate (4–6)	112 (29.2)	61 (28.8)	51 (29.7)
High (7–10)	109 (28.4)	64 (30.2)	45 (26.2)
At risk stress level *	52 (13.6)	32 (15.1)	20 (11.7)
Sources of stress			
Work	151 (39.3)	92 (43.4)	59 (34.3)
Finances	115 (29.9)	68 (32.1)	47 (27.3)
Language barriers	31 (8.1)	15 (7.1)	16 (9.3)
Family	68 (17.7)	30 (14.2)	38 (22.1)
Health	68 (17.7)	31 (14.6)	37 (21.5)
Coping strategies ^#^			
Sleeping or resting	154 (40.1)	86 (40.6)	68 (39.5)
Talking to someone	85 (22.1)	35 (16.5)	50 (29.1)
Exercise	80 (20.8)	51 (24.1)	29 (16.9)
Religious or spiritual practice	22 (5.7)	16 (7.5)	6 (3.5)
Other	15 (3.9)	9 (4.2)	6 (3.5)

Data are presented as numbers and percentages. * At-risk stress level was defined as reporting feeling stressed ‘often’ and having a self-reported stress level ≥ 7 on the 1–10 scale. ^#^ A statistically significant difference between sexes was observed only for the ‘Coping strategies’ category based on the Chi-square test, as indicated by # (*p* = 0.017).

**Table 7:** Anthropometric characteristics and obesity prevalence.

	Female	Male	*p*-Value
BMI * (Median, IQR)	20.03 (4.26)	23.13 (4.50)	<0.001
WC ^#^ (Median, IQR)	67.0 (15.5)	80.0 (12.0)	<0.001
General obesity, *n* (%) *			<0.001
Underweight	38 (22.1)	11 (5.2)	
Normal weight	111 (64.5)	136 (64.2)	
Overweight	16 (9.3)	47 (22.2)	
Obesity	7 (4.1)	18 (8.5)	
Abdominal obesity, ^#^ *n* (%)			
WC—Increased risk ^$^	19 (11.3)	17 (8.1)	0.162
WC—Substantially increased risk ^$^	6 (3.6)	16 (7.6)	

* Number of respondents: Female-172, Male-212, ^#^ Number of respondents: Female-158, Male-200, ^$^ Increased risk refers to WC > 94 cm in men and >80 cm in women. A substantially increased risk refers to WC > 102 cm and >88 cm in women [40].

**Table 8:** Co-occurrence of risk factors by age and sex.

No. of Risk Factors	Age Group		
	30–39 (*n* = 116)	40–49 (*n* = 149)	50–60 (*n* = 119)
Female (*n* = 172)			
1	2 (4.3)	5 (7.6)	2 (3.4)
2	16 (34.0)	18 (27.3)	12 (20.3)
3	12 (25.5)	17 (25.8)	21 (35.6)
4	12 (25.5)	13 (19.7)	19 (32.2)
5	4 (8.5)	9 (13.6)	5 (8.5)
6	1 (2.1)	4 (6.1)	0
Male (*n* = 212)			
1	4 (5.7)	2 (2.4)	3 (5.1)
2	11 (15.7)	9 (10.8)	7 (11.9)
3	31 (44.3)	29 (34.9)	13 (22.0)
4	15 (21.4)	25 (30.1)	20 (33.9)
5	8 (11.4)	12 (14.5)	11 (18.6)
6	1 (1.4)	6 (7.2)	3 (5.1)
7	0	0	2 (3.4)

Data are presented as numbers and percentages (calculated within each age group). The number of risk factors refers to the total number of NCD risk factors (tobacco use, harmful alcohol consumption, inadequate intake of fruits and vegetables, frequent consumption of UPF, insufficient physical activity, high BMI, high stress level, and lack of sleep) present in each individual.

**Table 9:** Participant characteristics and association with latent class membership.

		Tobacco/Alcohol-Diet Cluster (*n* = 180)	Sedentary Life-Diet Cluster (*n* = 204)	*p*-Value
Age	30–39 years	54 (30.0)	63 (30.9)	0.980
	40–49 years	70 (38.9)	79 (38.7)	
	50–60 years	56 (31.1)	62 (30.4)	
Sex	Male	123 (68.3)	89 (43.6)	<0.001
	Female	57 (31.7)	115 (56.4)	
Employment status	Company employee (Full-time)	102 (56.7)	91 (44.6)	0.021
	Company employee (Contract)	12 (6.7)	15 (7.4)	
	Part-time work	20 (11.1)	33 (16.2)	
	Government employee	14 (7.8)	5 (2.5)	
	Self-employee	4 (2.2)	9 (4.4)	
	Housewife	7 (3.9)	24 (11.8)	
	Business owner/executive	4 (2.2)	5 (2.5)	
	Doctor/medical personnel	3 (1.7)	1 (0.5)	
	Freelancer	4 (2.2)	5 (2.5)	
	Unemployed	7 (3.9)	7 (3.4)	
	Student	1 (0.6)	1 (0.5)	
	Other	2 (1.1)	7 (3.4)	
Annual household income	<1,000,000¥	9 (5.0)	10 (4.9)	0.823
	1,000,000–4,999,999¥	57 (31.7)	76 (37.3)	
	5,000,000–9,999,999¥	77 (37.7)	81 (39.7)	
	10,000,000–14,999,999¥	24 (13.3)	20 (9.8)	
	15,000,000–19,999,999¥	9 (5.0)	9 (4.4)	
	>20,000,000¥	4 (2.2)	8 (3.9)	
Degree of Education	No formal schooling	2 (1.1)	4 (2.0)	0.180
	Primary/elementary school	4 (2.2)	2 (1.0)	
	Secondary/junior high school	6 (3.3)	8 (3.9)	
	High school	57 (31.7)	44 (21.6)	
	College/university	89 (49.4)	105 (51.5)	
	Postgraduate	21 (11.7)	39 (19.1)	
	Other	1 (0.6)	2 (1.0)	
Living condition	Living alone	50 (27.8)	50 (24.5)	0.398
	Living with spouse/partner	39 (21.7)	45 (22.1)	
	Living with family	89 (49.4)	109 (53.4)	
	Other	2 (1.1)	0	
Presence of medical conditions	High blood pressure	45 (25.0)	24 (11.8)	<0.001
	Diabetes	18 (10.0)	9 (4.4)	0.033
	High cholesterol	41 (22.8)	26 (12.7)	0.010
	Heart diseases	18 (10.0)	4 (2.0)	<0.001

Data are presented as numbers and percentages (calculated within each cluster). Pearson’s chi-square test was used to assess differences between clusters.

## Data Availability

The data presented in this study are available on a reasonable request from the corresponding author due to privacy and ethical reasons.

## Supplementary Materials

The following supporting information can be downloaded at: https://www.mdpi.com/article/10.3390/healthcare13212781/s1, Supplementary Document: Supplementary Tables. Table S1: STROBE reporting checklist for cross-sectional study; Table S2: Fit indices for latent class models (K = 2–10); Table S3: Patterns, duration, and frequency of cigarette, e-cigarette, chewable tobacco, and alcohol use; Table S4: Current use of alcohol and tobacco products by sex; Table S5: Fruit and vegetable consumption among participants; Table S6: Adequacy of fruit and vegetable intake by sex; Table S7: Self-reported frequency of consumption of ultra-processed foods (*n* = 384); Table S8: Physical activity habits by sex; Table S9: Co-occurrence of NCD risk factors by participant characteristics.

## Abbreviations

The following abbreviations are used in this manuscript:

BMI	Body mass index
NCDs	Non-communicable diseases
LCA	Latent class analysis
PA	Physical activity
UPF	Ultra-processed foods
WC	Waist circumference
