# Supplementary material for: Prevalence and Clustering of Lifestyle Risk Factors for Chronic Diseases Among Middle-Aged Migrants in Japan
Source: Healthcare (Basel). 2025 Nov 2;13(21):2781. doi: 10.3390/healthcare13212781 (PMC12610008; doi:10.3390/healthcare13212781)
Supplement: Supplementary file 1 [file healthcare-13-02781-s001.zip › healthcare-3898660-supplementary.pdf]

Supplementary Tables

Table S1 – STROBE reporting checklist for cross-sectional study.

		Reporting Item	Page Number
**Title and abstract**			
Title	#1a	Indicate the study's design with a commonly used term in the title or the abstract	1
Abstract	#1b	Provide in the abstract an informative and balanced summary of what was done and what was found	1
**Introduction**			
Background / rationale	#2	Explain the scientific background and rationale for the investigation being reported	1-3
Objectives	#3	State specific objectives, including any prespecified hypotheses	2-3
**Methods**			
Study design	#4	Present key elements of study design early in the paper	3
Setting	#5	Describe the setting, locations, and relevant dates, including periods of recruitment, exposure, follow-up, and data collection	3-6
Eligibility criteria	#6a	Give the eligibility criteria, and the sources and methods of selection of participants.	3
	#7	Clearly define all outcomes, exposures, predictors, potential confounders, and effect modifiers. Give diagnostic criteria, if applicable	
Data sources / measurement	#8	For each variable of interest give sources of data and details of methods of assessment (measurement). Describe comparability of assessment methods if there is more than one group. Give information separately for for exposed and unexposed groups if applicable.	3-6
Bias	#9	Describe any efforts to address potential sources of bias	2-3
Study size	#10	Explain how the study size was arrived at	3
Quantitative variables	#11	Explain how quantitative variables were handled in the analyses. If applicable, describe which groupings were chosen, and why	6
Statistical methods	#12a	Describe all statistical methods, including those used to control for confounding	6
Statistical methods	#12b	Describe any methods used to examine subgroups and interactions	6
Statistical methods	#12c	Explain how missing data were addressed	4-5

Statistical methods	#12d	If applicable, describe analytical methods taking account of sampling strategy	n/a
Statistical methods	#12e	Describe any sensitivity analyses	n/a
**Results**			
Participants	#13a	Report numbers of individuals at each stage of study—eg numbers potentially eligible, examined for eligibility, confirmed eligible, included in the study, completing follow-up, and analysed. Give information separately for for exposed and unexposed groups if applicable.	3-4
Participants	#13b	Give reasons for non-participation at each stage	3-4
Participants	#13c	Consider use of a flow diagram	n/a
Descriptive data	#14a	Give characteristics of study participants (eg demographic, clinical, social) and information on exposures and potential confounders. Give information separately for exposed and unexposed groups if applicable.	7-8
Descriptive data	#14b	Indicate number of participants with missing data for each variable of interest	7-15 (Valid no.of responses has mentioned)
Outcome data	#15	Report numbers of outcome events or summary measures. Give information separately for exposed and unexposed groups if applicable.	7-15
Main results	#16a	Give unadjusted estimates and, if applicable, confounder-adjusted estimates and their precision (eg, 95% confidence interval). Make clear which confounders were adjusted for and why they were included	n/a
Main results	#16b	Report category boundaries when continuous variables were categorized	7-15
Main results	#16c	If relevant, consider translating estimates of relative risk into absolute risk for a meaningful time period	n/a
Other analyses	#17	Report other analyses done—e.g., analyses of subgroups and interactions, and sensitivity analyses	n/a
**Discussion**			
Key results	#18	Summarise key results with reference to study objectives	16
Limitations	#19	Discuss limitations of the study, taking into account sources of potential bias or imprecision. Discuss both direction and magnitude of any potential bias.	20
Interpretation	#20	Give a cautious overall interpretation considering objectives, limitations, multiplicity of analyses, results from similar studies, and other relevant evidence.	16-20
Generalisability	#21	Discuss the generalisability (external validity) of the study results	20 (under limitations)
**Other Information**			

Funding	#22	Give the source of funding and the role of the funders for the present study and, if applicable, for the original study on which the present article is based	21

Table S2. Fit indices for latent class models (K=2-10)

Number of classes (K)	Log-likelihood	AIC	BIC
2	−1643.0	3320.0	**3387.0**
3	−1635.0	3323.0	3426.0
4	−1629.0	3328.0	3467.0
5	−1624.0	3337.0	3511.0
6	−1619.0	3345.0	3554.0
7	−1614.0	3352.0	3597.0
8	−1610.0	3361.0	3642.0
9	−1606.0	3372.0	3688.0
10	−1602.0	3383.0	3734.0

Table S3. Patterns, duration, and frequency of cigarette, e-cigarette, chewable tobacco, and alcohol use

		Cigarettes	E-cigarettes	Chewable tobacco	Alcohol
Practice (n=384)	Current users	70 (18.2)	55 (14.3)	11 (2.9)	164 (42.7)
	Former users	109 (28.4)	77 (20.1)	44 (11.5)	90 (23.4)
	Never used	205 (53.4)	252 (65.6)	329 (85.7)	130 (33.9)
Duration of use (current users)#	<6 months	9 (12.9)	8 (14.5)	2 (18.2)	35 (21.3)
	6 months-1 year	5 (7.1)	2 (3.6)	6 (54.5)	5 (3.0)
	>1year - 5 years	4 (5.7)	15 (27.3)	2 (18.2)	6 (3.7)
	>5 years	52 (74.3)	30 (54.5)	1 (9.1)	118 (72.0)
Duration of use (former users) #	<6 months	11 (10.1)	18 (23.4)	5 (11.4)	21 (23.3)
	6 months-1 year	23 (21.1)	22 (28.6)	13 (29.5)	19 (21.1)
	>1year - 5 years	22 (20.2)	26 (33.8)	19 (43.2)	19 (21.1)
	>5 years	53 (48.6)	11 (14.3)	7 (15.9)	31 (34.3)
Units per day* (current users) #	1-2	9 (12.9)	7 (12.7)	5 (45.5)	104 (63.4)
	3-5	9 (12.9)	11 (20.0)	3 (27.3)	38 (23.2)
	6-10	21 (30.0)	16 (29.1)	3 (27.3)	10 (6.1)
	>11	31 (44.3)	21 (38.2)	0 (0)	12 (7.3)
Units per day* (former users)#	1-2	39 (35.8)	32 (41.6)	14 (31.8)	57 (63.3)
	3-5	31 (28.4)	27 (35.1)	17 (38.6)	22 (24.4)
	6-10	17 (15.6)	7 (9.1)	8 (18.2)	5 (5.6)
	>11	22 (20.2)	11 (14.3)	5 (11.4)	6 (6.7)
Unit x Duration (Current users) #	>1 year, 3-5/ day	5 (7.1)	7 (12.7)	1 (9.1)	31 (18.9)
	>1 year, 6-10/ day	17 (24.3)	16 (29.0)	0	8 (4.9)
	>1 year, >10/ day	28 (40.0)	19 (34.6)	0	9 (5.5)
	>5 year, 3-5/ day	3 (4.3)	4 (4.3)	1 (9.1)	29 (17.7)
	>5 year, 6-10/ day	22 (31.4)	11 (20.0)	0	8 (4.9)
	>5 year, >10/ day	23 (32.9)	12 (21.8)	0	9 (5.5)
Unit x Duration (former users) #	>1 year, 3-5/ day	12 (11.0)	11 (14.3)	11 (25.0)	10 (11.1)
	>1 year, 6-10/ day	14 (12.8)	5 (6.5)	4 (9.1)	2 (2.2)
	>1 year, >11/ day	20 (18.4)	7 (9.1)	5 (11.4)	4 (4.4)
	>5 year, 3-5/ day	2 (1.8)	2 (2.6)	1 (2.3)	3 (3.3)
	>5 year, 6-10/ day	10 (9.2)	4 (5.2)	1 (2.3)	1 (1.1)

	>5 year, >11/ day	18 (16.5)	3 (3.9)	3 (6.8)	4 (4.4)

Data are presented as numbers and percentages. *Units per day refers to the number of cigarettes smoked per day, number of sessions or puffs of e-cigarettes taken per day, number of times chewable tobacco is used per day, and number of alcoholic drinks or shots taken per day. # Percentages were calculated based on the respective number of current or former users for each product.

Table S4. Current use of alcohol and tobacco products by sex

	Female (n=172)	Male (n=212)	Total (n=384)	p-value
Only alcohol	36 (20.9)	68 (32.1)	104 (27.1)	0.0199
Cigarettes only	6 (3.5)	14 (6.6)	20 (5.2)	0.2562
E-cigarettes only	5 (2.9)	9 (4.2)	14 (3.6)	0.673
Alcohol & Cigarettes	6 (3.5)	13 (6.1)	19 (4.9)	0.3414
Alcohol & E-cigarettes	3 (1.7)	9 (4.2)	12 (3.1)	0.2688
Alcohol & chewable tobacco	0 (0.0)	3 (1.4)	3 (0.8)	0.3254
Cigarettes & E-cigarettes	1 (0.6)	3 (1.4)	4 (1.0)	0.7681
Cigarettes & chewable tobacco	1 (0.6)	0 (0.0)	1 (0.3)	0.9165
Alcohol, Cigarettes E-cigarettes	7 (4.1)	13 (6.1)	20 (5.2)	0.5006
All four	1 (0.6)	5 (2.4)	6 (1.6)	0.3258

Data are presented as numbers and percentages, except for p-values. p-values are based on Chi-square tests. A statistically significant sex difference was observed only for the "Only alcohol" category.

Table S5. Fruit and vegetable consumption among participants

		**Fresh fruits**	**Green veg.**	**Legumes**	**Roots & Tubers#**	**Other vegetables**
Consumption	Everyday	71 (18.5)	96 (25.0)	40 (10.4)	48 (12.5)	63 (16.4)
	5-6 days	46 (12.0)	71 (18.5)	42 (10.9)	58 (15.1)	74 (19.3)
	3-4 days	75 (19.5)	103 (26.8)	77 (20.1)	85 (22.1)	101 (26.3)
	1-2 days	73 (19.0)	53 (13.8	86 (22.4)	100 (13.5)	77 (20.1)
	Once/ week	45 (11.7)	31 (8.1)	66 (17.2)	41 (17.2)	37 (9.6)
	Rarely/ never	74 (19.3)	30 (7.8)	73 (19.0)	73 (10.7)	32 (8.3)
Number of servings per day*	<1 servings	113 (29.4)	116 (30.2)	124 (32.3)	125 (32.6)	123 (32.0)
	1 serving	111 (28.9)	129 (33.6)	106 (27.6)	126 (32.8)	126 (32.8)
	2 servings	65 (16.9)	80 (20.8)	52 (13.5)	63 (16.4)	72 (18.8)
	3 servings	17 (4.4)	22 (5.7)	26 (6.8)	23 (6.0)	24 (6.3)
	>4 servings	4 (1.0)	7 (1.8)	3 (0.8)	6 (1.6)	7 (1.8)

#"Roots and tubers" refer to edible non-starchy roots (carrots, beets, radishes, turnips) and exclude starchy root crops such as potatoes, cassava, and yams. *1 count of serving refers to '1 cup of raw vegetables,' '½ cup of cooked vegetables,' '1 medium-sized fruit,' and '1/2 cup of cut fruit.'

Table S6. Adequacy of fruit and vegetable intake by sex

	Female (n=172)	Male (n=212)	Total (n=384)	p-value
Sufficient fruit intake	12 (7.0)	6 (2.8)	18 (4.7)	0.0951
Sufficient vegetable intake	47 (27.3)	89 (19.8)	20 (23.2)	0.1066
Sufficient fruit & veg. intake	11(5.8)	4 (1.4)	15 (3.9)	0.0452

Values represent numbers and percentages of participants who met the recommended intake of fruits (>2 servings) and vegetables (>3 servings) within each sex. P-values are based on Chi-square tests. A statistically significant sex difference was observed only for sufficient fruit and vegetable intake.

Table S7. Self-reported frequency of consumption of ultra-processed foods (n=384)

	Processed salty foods	Sugary drinks	Sweet snacks	Deep-fried food	High-fat processed foods
Almost everyday	22 (5.7)	54 (14.1)	32 (8.3)	16 (4.2)	31 (8.1)
5-6 days/ week	34 (8.9)	31 (8.1)	42 (10.9)	27 (7.0)	23 (6.0)
3-4 days/ week	59 (15.4)	59 (15.4)	68 (17.7)	55 (14.3)	65 (16.9)
1-2 days/ week	105 (27.3)	88 (22.9)	111 (28.9)	136 (35.4)	124 (32.3)
2-3 times/ months	52 (13.5)	39 (10.2)	63 (16.4)	68 (17.7)	66 (17.2)
Once/ month	39 (10.2)	36 (9.4)	33 (8.6)	45 (11.7)	35 (9.1)
Rarely/ never	73 (19.0)	77 (20.1)	35 (9.1)	37 (9.6)	40 (10.4)

Data are presented as numbers and percentages.

Table S8. Physical activity habits by sex

	Female (n=172)	Male (n=212)	p-value
Moderate PA	87 (50.6)	112 (52.8)	0.661
Vigorous PA	44 (25.6)	87 (41.0)	0.001
Sports/ fitness	59 (34.3)	99 (46.7)	0.014
Adequate PA*	75 (43.6)	120 (56.6)	0.011

Data are presented as numbers and percentages. *Adequate PA (>150 minutes/ week moderate PA, >75 minutes per week vigorous PA, or an equivalent combination). P-values are based on Chi-square tests. A statistically significant sex difference was observed for vigorous PA, sports/ fitness, and adequate PA. PA- Physical activity.

Table S9. Co-occurrence of NCD risk factors by participant characteristics

		1 (n=18)	2 (n=73)	3 (n=123)	4 (n=104)	5 (n=49)	6 (n=15)	7 (n=2)
Age	30-39 years	6 (33.3)	27 (37.0)	43 (35.0)	27 (26.0)	12 (24.5)	2 (13.3)	0 (0.0)
	40-49 years	7 (38.9)	27 (37.0)	46 (37.4)	38 (36.5)	21 (42.9)	10 (66.7)	0 (0.0)
	50-60 years	5 (27.8)	19 (26.0)	34 (27.6)	39 (37.5)	16 (32.7)	3 (20.0)	2 (100.0)
Sex*	Male	9 (50.0)	46 (63.0)	50 (40.7)	44 (42.3)	18 (36.7)	5 (33.3)	0 (0.0)
	Female	9 (50.)	27 (37.0)	73 (59.3)	60 (57.7)	31 (63.3)	10 (66.7)	2 (100.0)
Employment status	Company employee (Full time)	8 (44.4)	32 (43.8)	60 (48.8)	60 (57.7)	28 (57.1)	3 (20.0)	2 (100.0)
	Company employee (Contract)	0 (0.0)	5 (6.8)	14 (11.4)	4 (3.8)	1 (2.0)	3 (20.0)	0 (0.0)
	Part-time work	1 (5.6)	15 (20.5)	17 (13.8)	10 (9.6)	7 (14.3)	3 (20.0)	0 (0.0)
	Government employee	0 (0.0)	3 (4.1)	4 (3.3)	8 (7.7)	3 (6.1)	1 (6.7)	0 (0.0)
	Self-employee	2 (11.1)	1 (1.4)	3 (2.4)	4 (3.8)	2 (4.1)	1 (6.7)	0 (0.0)
	Housewife	2 (11.1)	10 (13.7)	8 (6.5)	6 (5.8)	4 (8.2)	1 (6.7)	0 (0.0)
	Business owner/ executive	1 (5.6)	1 (1.4)	3 (2.4)	2 (1.9)	1 (2.0)	0 (0.0)	0 (0.0)
	Doctor/ medical personnel	0 (0.0)	1 (1.4)	1 (0.8)	2 (1.9)	0 (0.0)	0 (0.0)	0 (0.0)
	Freelancer	2 (11.1)	1 (1.4)	3 (2.4)	1 (1.0)	0 (0.0)	2 (13.3)	0 (0.0)
	Unemployed	0 (0.0)	0 (0.0)	7 (5.7)	3 (2.9)	3 (6.1)	1 (6.7)	0 (0.0)
	Student	0 (0.0)	1 (1.4)	1 (1.4)	0 (0.0)	0 (0.0)	0 (0.0)	0 (0.0)
	Other	2 (11.1)	3 (4.1)	2 (1.6)	2 (1.9)	0 (0.0)	0 (0.0)	0 (0.0)
Annual	<1,000,000¥	1 (5.6)	1 (1.4)	9 (7.3)	3 (2.9)	3 (6.1)	2 (13.3)	0 (0.0)

household income	1,000,000 – 4,999,999¥	9 (50.0)	25 (34.2)	47 (38.2)	33 (31.7)	18 (36.7)	9 (60.0)	1 (50.0)
	5,000,000 – 9,999,999¥	9 (50.0)	30 (41.1)	50 (40.7)	43 (41.3)	22 (44.9)	8 (26.7)	0 (0.0)
	10,000,000 - 14,999,999¥	2 (11.1)	9 (12.3)	15 (12.2)	15 (14.4)	4 (8.2)	0 (0.0)	0 (0.0)
	15,000,000 - 19,999,999¥	1 (5.6)	6 (8.2)	14 (11.4)	9 (8.7)	2 (4.1)	1 (6.7)	1 (50.0)
	>20,000,000¥	0 (0.0)	2 (2.7)	2 (1.6)	1 (1.0)	0 (0.0)	0 (0.0)	0 (0.0)
Degree of Education	No formal schooling	0 (0.0)	0 (0.0)	4 (3.3)	1 (1.0)	0 (0.0)	1 (6.7)	0 (0.0)
	Primary/ elementary school	0 (0.0)	2 (2.7)	2 (1.6)	1 (1.0)	0 (0.0)	1 (6.7)	0 (0.0)
	Secondary/ junior high school	0 (0.0)	2 (2.7)	3 (2.4)	5 (4.8)	2 (4.1)	2 (13.3)	0 (0.0)
	High school	1 (5.6)	13 (17.8)	31 (25.2)	28 (26.9)	22 (44.9)	5 (33.3)	1 (50.0)
	College/ university	13 (72.2)	48 (65.8)	55 (44.7)	52 (50.0)	20 (40.8)	5 (33.3)	1 (50.0)
	Postgraduate	3 (16.7)	7 (9.6)	27 (22.0)	17 (16.3)	5 (10.2)	1 (6.7)	0 (0.0)
	Other	1 (5.6)	1 (1.4)	1 (0.8)	0 (0.0)	0 (0.0)	0 (0.0)	0 (0.0)
Living condition	Living alone	7 (38.9)	16 (21.9)	30 (24.4)	24 (23.1)	18 (36.7)	5 (33.3)	0 (0.0)
	Living with spouse/ partner	3 (16.7)	21 (28.8)	29 (23.6)	22 (21.2)	6 (12.2)	3 (20.0)	0 (0.0)
	Living with family	8 (44.4)	36 (49.3)	63 (51.2)	58 (55.8)	25 (51.0)	6 (40.0)	2 (100.0)
	Other	0 (0.0)	0 (0.0)	1 (0.8)	0 (0.0)	0 (0.0)	1 (6.7)	0 (0.0)
Presence of medical conditions	High blood pressure*	1 (5.6)	9 (12.3)	16 (13.0)	22 (21.2)	14 (28.6)	6 (40.0)	1 (50.0)
	Diabetes*	1 (5.6)	3 (4.1)	6 (4.9)	6 (5.8)	8 (16.3)	2 (13.3)	1 (50.0)
	High cholesterol*	0 (0.0)	5 (6.8)	19 (15.4)	25 (24.0)	11 (22.4)	6 (40.0)	1 (50.0)
	Heart diseases	0 (0.0)	5 (6.8)	5 (4.1)	6 (5.8)	5 (10.2)	1 (6.7)	0 (0.0)

Data are presented as numbers and percentages.
